# Identification and analysis of *in planta *expressed genes of *Magnaporthe oryzae*

**DOI:** 10.1186/1471-2164-11-104

**Published:** 2010-02-10

**Authors:** Soonok Kim, Jongsun Park, Sook-Young Park, Thomas K Mitchell, Yong-Hwan Lee

**Affiliations:** 1 Department of Agricultural Biotechnology, Center for Fungal Pathogenesis, Center for Agricultural Biomaterials and Center for Fungal Genetic Resources, Seoul National University, Seoul 151-921, Korea; 2 Department of Plant Pathology, The Ohio State University, Columbus, OH 43210, USA

## Abstract

**Background:**

Infection of plants by pathogens and the subsequent disease development involves substantial changes in the biochemistry and physiology of both partners. Analysis of genes that are expressed during these interactions represents a powerful strategy to obtain insights into the molecular events underlying these changes. We have employed expressed sequence tag (EST) analysis to identify rice genes involved in defense responses against infection by the blast fungus *Magnaporthe oryzae *and fungal genes involved in infectious growth within the host during a compatible interaction.

**Results:**

A cDNA library was constructed with RNA from rice leaves (*Oryza sativa *cv. Hwacheong) infected with *M. oryzae *strain KJ201. To enrich for fungal genes, subtraction library using PCR-based suppression subtractive hybridization was constructed with RNA from infected rice leaves as a tester and that from uninfected rice leaves as the driver. A total of 4,148 clones from two libraries were sequenced to generate 2,302 non-redundant ESTs. Of these, 712 and 1,562 ESTs could be identified to encode fungal and rice genes, respectively. To predict gene function, Gene Ontology (GO) analysis was applied, with 31% and 32% of rice and fungal ESTs being assigned to GO terms, respectively. One hundred uniESTs were found to be specific to fungal infection EST. More than 80 full-length fungal cDNA sequences were used to validate *ab initio* annotated gene model of *M. oryzae* genome sequence.

**Conclusion:**

This study shows the power of ESTs to refine genome annotation and functional characterization. Results of this work have advanced our understanding of the molecular mechanisms underpinning fungal-plant interactions and formed the basis for new hypothesis.

## Background

Rice blast, caused by *Magnaporthe oryzae*, is one of the most devastating diseases in rice growing regions worldwide, causing 11-15% yield loss annually [1]. Genetic tractability as well as economic importance makes this disease a model pathosystem to understand plant-microbe interactions. The genome sequences of both organisms are available [2-4], and both forward and reverse genomic studies to understand molecular mechanisms for pathogenesis on a genome scale have been undertaken [5]. Understanding the precise molecular mechanisms of infection will facilitate design of novel control strategies.

The process of *M. oryzae *infection starts when a conidium lands on the rice leaf surface. After germination by hydration, an appressorium develops at the tip of the germ tube, from which a penetration peg emerges to penetrate the cuticle layer into the rice cell using mechanical force. Within the plant cell, the fungus faces two different fates. In an incompatible interaction, resistance gene products recognize corresponding avirulence gene products from the invading pathogen and invoke a series of defense responses to restrict pathogen growth [6]. In a compatible interaction, however, the host plant mobilizes defense responses much later, resulting in visible coalescing lesions. From these expanding lesions, a conidiophore emerges, releasing tens of thousands of new conidia able to start a second round of infection. In this respect, the gene products of the pathogen expressed during infectious growth may play roles as pathogenicity factors required for evasion of the host's defense responses and successful colonization. This theory is supported by our previous pilot study in which analysis of *in planta *expressed genes identified by EST sequencing of an *M.oryzae *infected rice cDNA library [7]. By sequencing 511 randomly selected cDNA clones, 72 of 293 ESTs could be assigned as fungal genes with sequence similarity to NCBI entries. Among them, *MHP1*, encoding a Class II hydrophobin, was preferentially expressed during infectious growth and was involved in full virulence of rice blast fungus, acting in the late stages of infection [8]. We also identified novel pathogenesis-related protein genes that are upregulated in rice after pathogen infection [9,10]. Jantasuriyarat et al. [11] performed large-scale analysis of ESTs during *M.oryzae*/rice interactions at the early stage of infection. Plant defense genes were well represented in this study. However, fungal genes involved in interactions with the host were scarce, primarily because they harvested infected tissue at 6 and 24 h after inoculation, at which time fungal spores had just started to penetrate. Genome-wide transcriptome analyses have also been conducted on this fungus within the last few years through the use of EST, SAGE, and RL-SAGE [7,11-16]. Most of these studies focused on pre-penetration stages, such as conidiation and appressorium formation, and on growth in a variety of *in vitro *conditions, including complete medium, minimal medium, nitrogen starvation, and rice cell wall medium. From these global efforts, 28,682 EST sequences comprising 8,821 uniESTs of *M. oryzae *are registered in the dbEST of NCBI, which covers about 80% of the genes electronically annotated for this fungus [2]. However, our knowledge of the molecular basis of the establishment of disease and proliferation within the plant cells remains limited.

During recent years, studies aimed at cataloging pathogen genes expressed during interactions with their host have been conducted in many pathosystems. Large-scale EST analysis with cDNA libraries from infected plant tissues has been used for this purpose [17]. More than 1,000 fungal genes could be identified out of 1,869 EST sequences from senescent leaves (21-25 days after inoculation) with lesions containing visible pycnidia. However, few fungal genes could be recovered from *Fusarium graminearum *infected wheat leaves (86 of 3,546 uniESTs) [18], from *Brassica napus *stem segments with expanding lesions caused by *Sclerotinia sclerotiorum *(52 of 767 uniESTs) [19], from wheat roots infected by take-all fungus (9 of 114 ESTs) [20], or *M.oryzae *infected rice leaves (4 of 13,570 uniESTs) [11]. Low-level representation of fungal genes may be because they harvested infected tissues at early time points. Suppression subtractive hybridization was also applied to identify genes expressed during the pathogen's interaction with its host [20,21]. Broeker et al. [21] identified 81 *Puccinia graminis *f. sp. *tritici *genes by sequencing 454 random clones from rust-infected wheat plants subtracted from those of healthy leaves. In this study, we used two complementary approaches: EST analysis and suppression subtractive hybridization and subsequent sequencing to catalog genes expressed during rice-*M.oryzae *interactions at the genome level. 712 fungal genes were identified from 2,315 uniESTs and their putative functions were assessed with GO. We were able to identify 100 novel fungal genes specific to infection. cDNAs with full-length ORFs were used to correct *ab initio *gene models annotated from genome sequences, and to analyze fungal splice site context.

## Results

### Generation of infection ESTs

We identified *in planta *expressed fungal genes by EST analysis of a *M.oryzae*-infected rice cDNA library in a previous study [7]. Seventy-two ESTs comprising 19 unigenes were assigned as fungal genes. To expand our knowledge of the molecular mechanisms underlying plant-microbe interactions, cataloging gene repertoires at a genome-wide level was carried out with two complementary strategies. First, an infection library (IL) was constructed with RNAs from leaves showing rapidly expanding lesions. cDNAs more than 0.5 kb in size after gel filtration were used for library construction. The titer of the primary library was 1.85 × 10^7^pfu/μg vector, and the insert size ranged from 0.5 to 2.0 kb. Sequencing of 1,976 randomly selected cDNAs generated 1,539 non-redundant ESTs, of which 654 ESTs formed 217 contigs and 1,322 ESTs remained as singletons (Table 1). Second, to enrich for fungal genes expressed during colonization and propagation within the plant cells, as well as plant genes induced by the invading pathogen, we used suppression subtractive hybridization technology [22]. cDNAs synthesized with RNA from uninfected rice leaves were used as the driver, while those from heavily infected rice leaves were used as the tester. A total of 2,259 subtracted clones were sequenced, generating 963 nonredundant ESTs. In the subtraction library (SL), the redundancy of the sequences was relatively high, with 1,722 clones assembled into 426 contigs. When the sequences from two libraries were analyzed together, all the 4,235 ESTs fell into 2,302 non-redundant ESTs, in which 2,538 ESTs consisted of 605 contigs and 1,697 ESTs remained as singletons (Table 1). About 69% of these 2,302 nrESTs had high or moderate sequence similarity to the entries of the non-redundant database in NCBI through BLAST search.

**Table 1:** Summary of ESTs generated from infection and subtractrion libraries

Library	IL^1^	SL^2^	Total
Number of sequences analyzed	1,976	2,259	4,235
Number of contigs	217	426	605
Non-redundant EST	654	1,722	2,538
Number of singletons	1,322	537	1,697
Number of cDNAs contained in contigs	1,539	963	2,302

^1 ^IL means infection library constructed in UNI-ZAP XR vector.

^2 ^SL means subtraction library in which RNA from *M. oryzae *infected rice leaves was used as tester and that from uninfected leaves as driver.

The most frequently represented sequence was contig203, which showed sequence similarity to elongation factor 1-α of the dimorphic fungus *Ajellomyces capsulatus *[23]. It occurred 76 times from SL and five times from IL. Contig226 was sequenced 63 times only from SL, and Contig200, showing sequence similarity to senescence-associated protein of *Pisum sativum *[24], appeared 29 times in SL and once in IL. The repertoire of highly redundant clones differed between the two libraries (Additional file 1, Table S1). In IL, genes involved in photosynthesis, such as RuBisCo activase (Contig 526) and RuBisCo small subunit C (Contig 549), and plant defense-related genes, such as metallothionein (Contig 496) and pathogenesis-related protein 1 (Contig 597), were included in the 10 most redundant contigs. Only two contigs (Contig 310 and Contig 370) of the 10 most redundant contigs were considered to be of fungal origin. However, fungal genes made up 50% of the most redundant 10 contigs of SL. Additionally, after subtracting, genes involved in photosynthesis, defense-related genes including senescence-associated protein (Contig 200), probenazole-induced protein (Contig 173), and metallothionein I (Contig 153) were the most abundant sequences.

### Annotation of the origin of genes

**Figure 1:**
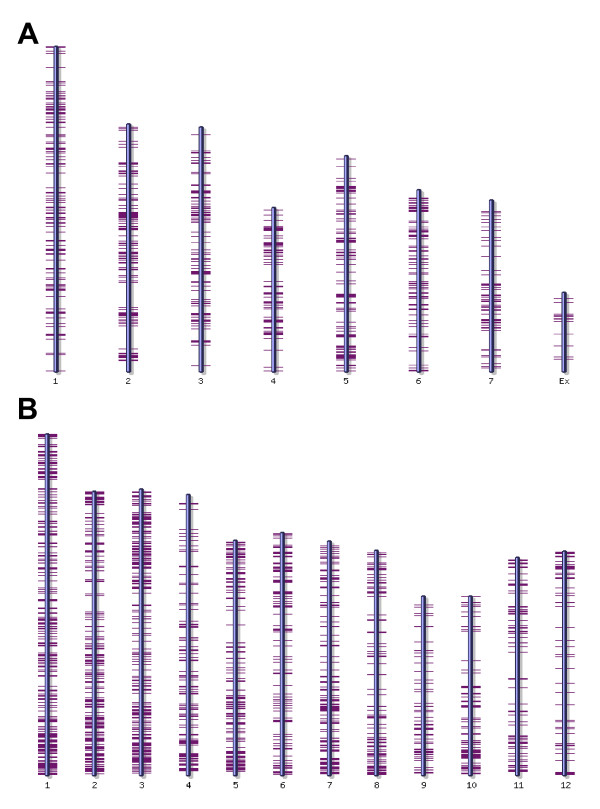
**Genome wide distribution of infection ESTs**. EST sequences were mapped to *M. oryzae *(A) or rice (B) chromosomes.

After initial analysis, the origin of ESTs was predicted. We took advantage of the most recent genome assembly of rice [3,4] and *M.oryzae *[2], as well as the deep cDNA sequence resources available for the host and pathogen. EST sequences were initially mapped on the genome sequence of both organisms, with 1,342 ESTs (773 UniESTs) being mapped to the fungal genome and 3,817 (1,546) to the rice genome (Fig. 1). Some of the ESTs were mapped to both genome sequences with the criteria we used (e<1e-5). Therefore, BLASTN data against 8,821 nrEST sequences from nine different libraries of *M. oryzae *[12], and 32,127 rice full-length cDNA (fl cDNA) sequences [25] and BLASTX data against NCBI non-redundant protein database were further analyzed together to assign the origin of uniESTs. As a result, 2,302 uniESTs could be grouped into 712 fungal genes (31%) and 1,562 plant genes (68%; Table 2). There were still 28 uniESTs of unidentified origin due to the lack of sequence similarity to either organism. The fungal genes consisted of 468 singletons and 244 contigs from 1,193 ESTs, whereas the rice genes consisted of 1,205 singletons and 357 contigs from 1,327 ESTs. ESTs from each library were evaluated in terms of origin of the genes. Of 1,539 uniESTs (1,976 ESTs) obtained from IL, 402 uniESTs (533 ESTs) could be assigned as fungal genes, which is 26.2% of the uniESTs (27% of total ESTs). For the 963 uniESTs of SL, 385 uniESTs, comprising 40% of uniESTs, were found to be of fungal origin. The proportion of fungal genes increased to 50% when the raw EST sequences were taken into consideration, as 1,128 ESTs of 2,259 ESTs from SL were grouped as fungal genes. Of the 712 uniESTs of fungal origin, 327 uniESTs were IL-specific and 310 uniESTs were only found in SL. Seventy-five genes were sequenced from both libraries. Similarly, 437 uniESTs from SL were added to the 1,125 rice genes identified from IL. We then questioned whether the fungal genes could cluster in the genome by aligning to the current genome assembly. However, infection ESTs did not cluster, but were rather spread evenly throughout the genome (Fig. 1).

**Table 2:** Classification of 2,315 uniESTs according to origin of the sequences

	Fungal genes	Rice genes	Unidentified
UniESTs (%*)	712 (30.9)	1,562 (67.9)	28 (1.2)
Singletons	468	1,205	24
Contigs (No. ESTs)	244 (1,193)	357 (1,327)	4 (48)

Infection library specific	327 (373)	1,038 (1,210)	
Both library	75 (587)	87 (568)	
Subtraction library specific	310 (701)	437 (754)	

The origin of sequences were determined by the searches of rice (Indica type) and *M. oryzae *(strain 70-15) draft genome sequence, dbEST with BLASTN algorithm at expect value of <e^-30 ^and NCBI non-redundant protein db with BLASTX algorithm (<e^-5^).

* Proportion was calculated based on 2,302 uniESTs.

### GO-based functional classification

**Figure 2:**
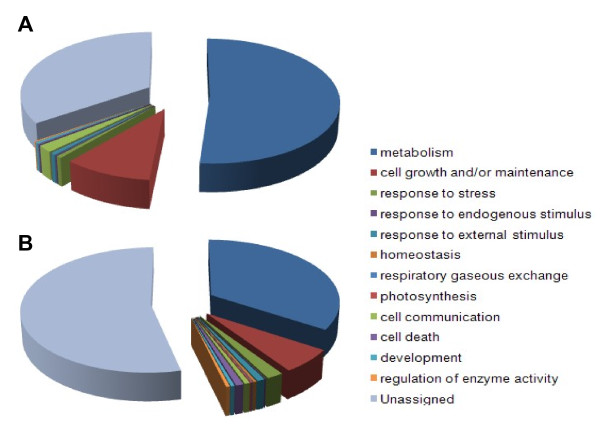
**Gene Ontology (GO) annotation of fungal (A) and plant genes (B) expressed during their interaction**. The ratio of hits to Biological Process is indicated. Fungal and rice ESTs were used to search matching ORFs from genome and full-length cDNA sequences, respectively. Translated ORF sequences were subjected to InterPro and GO analyses.

To assess the putative function of the ESTs, gene ontology (GO) based classification was conducted. Fungal and rice ESTs were translated in six frames, and the in-frame ORF sequences were subjected to InterPro and GO annotation, with 42.4% (663) of rice ESTs and 58.1% (414) of fungal ESTs being assigned GO terms. In terms of biological process, 463 rice and 285 fungal genes were assigned; most were associated with metabolism, followed by cell growth and/or maintenance. Nine uniESTs encoding fungal genes were assigned to cell communications (GO: 0007154), which contained two class II hydrophobins (F204 and F233), four GTP-binding proteins (F0250 encoding small GTPase RAS2, F0253, F0265, and F0694), and an NADP-dependent oxidoreductase (F0064). For molecular function, 556 rice and 343 fungal ESTs were assigned, most of which possessed binding and catalytic activity. In the 208 rice and 169 fungal ESTs assigned to cellular components, 199 rice and 165 fungal ESTs were associated with the cell, and were especially distributed within the intracellular and membrane (Fig. 2).

Proteins assigned as extracellular were poorly represented in this analysis, although secretion proteins are thought to play important roles in host interactions [26,27] and virulence [8,28,29]. Thus, we screened 2,302 uniESTs for signal peptides using SignalP3.0 [30]. Because most EST sequences did not bear a start codon, genomic resources were used. For fungal genes, *ab initio *annotated protein sequences matched to infection ESTs were subjected to SignalP analysis. In the case of rice genes, where 32,127 full-length (fl-) cDNA sequences are available, fl cDNA sequences matched to infection ESTs were translated and in-frame amino acid sequences were screened with SignalP. As a result, 63 fungal genes and 246 rice genes of 2,302 uniESTs were believed to have a signal peptide. Among these were three hydrophobins (F204, F233 encoding MHP1, and F238 encoding MPG1), two of which have been identified as secreted and associated with the cell wall [8,29]. ESTs encoding spore coat proteins (F207) and cell wall degrading enzymes, such as chitinase3 (F037) and 1,4-beta-D-glucan cellobiohydrolase (F298), were also identified.

### Search for novel genes uniquely or preferentially expressed during rice-*M. oryzae* interactions

**Figure 3:**
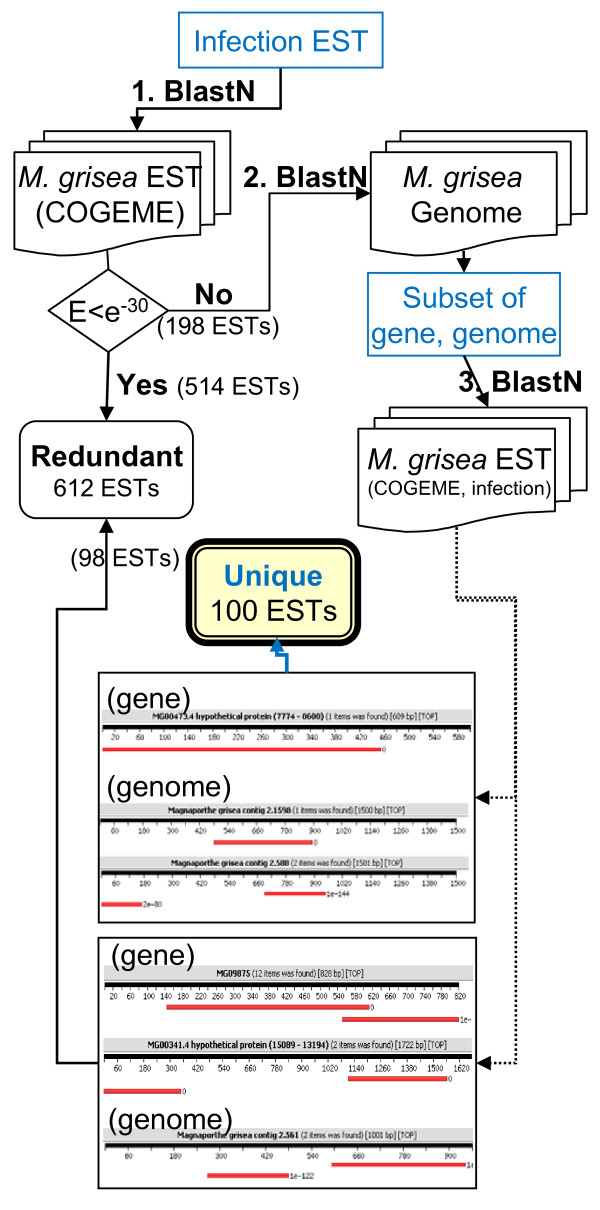
**Process to search novel genes uniquely or preferentially identified during rice-blast fungus interactions**.

We questioned whether there were novel genes as yet unidentified because the condition used (i.e., late stage of infection) is uncommon. To identify novel genes preferentially expressed during rice-*M. oryzae *interaction, 2,302 uniESTs were compared with EST or cDNA sequences of each organism using BLAST. *M. oryzae *uniEST sequences (8,820 COGEME_EST) were downloaded from COGEME phytopathogenic fungi and oomycete EST database (http://cogeme.ex.ac.uk). Rice full-length cDNA sequences (32,127) were downloaded from the KOME (Knowledge-based Oryza Molecular Biological Encyclopedia: http://cdna01.dna.affrc.go.jp/cDNA) website. For fungal genes, both COGEME EST and infectionEST collections were mapped on the *M. oryzae *genome, and the physical positions on the genome were compared between the two populations. Of the 712 putative fungal genes, 208 infectionESTs were not contained in the COGEME uniESTs. These EST sequences were further examined for sequences that represent different segments of the same gene present in other libraries to determine if they were truly novel genes. In the case of genic regions represented by two or more ESTs, these ESTs were considered to be the same gene. If two or more ESTs matched to non-genic sequences, they were connected end to end or placed with gaps less than 400 nts, these ESTs were also considered to represent the same gene (Fig. 3). Through this analysis, we finally assigned 100 fungal uniESTs (14%, 138 ESTs) as uniquely or preferentially expressed during infectious growth (Additional file 2, Table S2). Of these, 60 genes were not able to be assigned a putative function through the BLASTX search of NCBI non-redundant protein database. There were three groups of genes predicted to be functioning during direct interaction between the two organisms: genes involved in nutrient uptake and primary metabolism (F393, F415, F478, F561, F566, F575, and F597), cell wall processing (F287, F295, F305, and F541), and lipid metabolism (F125, F438, F553, and F636). Among these novel genes were two different members (F0438, MGG_05988 and F0553, MGG_09994) for phosphatidic acid phosphatase (PAP), which is an enzyme that deposphorylates phosphatidic acid (PA) to generate diacylglycerol (DAG), both of which are known to be involved in lipid metabolism and intracellular signal transduction [31,32]. The remaining member (MGG_09330) was sequenced from the nitrogen-starved library [12]. A gene (F0066, MGG_04205) homologous to avenacinase, which detoxifies plant saponins to determine the host range, was also identified as expressed *in planta*. With a similar approach, 52 rice uniESTs did not match the rice EST sequences registered in the dbEST of NCBI. Most of them showed no sequence similarity to the non-redundant protein database, suggesting a unique role(s) during interactions with the pathogen.

### Expression profiles of infection-enriched genes

**Figure 4:**
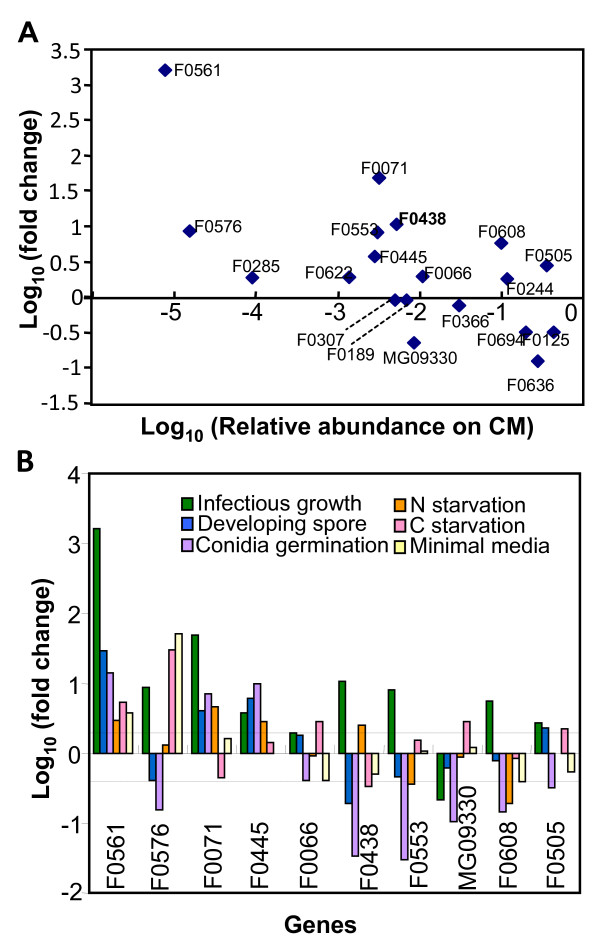
**Expression of infection EST specific fungal genes analyzed by real-time RT-PCR**. Relative abundance and fold changes were calculated as described in Experimental Procedures. Cyclophillin was used as an internal control. (A) Fold changes during infectious growth were compared relative abundance during growth in complete medium. (B) Fold changes during infectious growth, conidiation, conidial germination, and nutrient starvation conditions were depicted as log scale.

Expression profiles of 20 infection EST-specific genes were analyzed by real-time RT-PCR. Fold changes (2^-ΔΔCt^) during infectious growth were analyzed in terms of relative abundance (2^-ΔCt^) of transcripts during *in vitro *growth in complete media (CM), compared with the cyclophilin (CYP1) expression level (Fig. 4A). Analyzed genes were grouped into three categories according to the expression level in CM. Expression level of six genes were 0.1- to 0.45 fold that of CYP1 in CM; three were induced and three were repressed during infectious growth. Transcript levels of 10 genes were 0.1 to 3.0% that of CYP1 in CM; six of them were induced during *in planta *growth. Four genes were scarcely expressed in CM, but their expression was elevated *in planta*. Expression of nine genes upregulated more than twofold *in planta *was further examined during development and in nutrient conditions (Fig. 4B). Expression of F0561 encoding 3-phytase A was highly induced during infection and also induced more than twofold in all conditions tested. Expression of F0576 encoding aminopeptidase was co-up-regulated during infection, carbon starvation, and in minimal medium, while it was down-regulated during conidia germination. F0071, with unknown function, and F0445, encoding ATP-dependent RNA helicase, shared their expression pattern and were co-up-regulated during infection, asexual sporulation, conidia germination, and nitrogen starvation. The expression pattern of F0066, showing sequence similarity to avenacinase (*Gaeumannomyces graminis*), and F0505, homologous to MADS-box homolog *Umc1 *(*Ustilago maydis*), was the same, with induction *in planta*, in developing spores, and during carbon starvation, and repression in germination and minimal media. F0608, with sequence similarity to yeast maintenance protein dam1, had specific up-regulation during growth within the plant cell. Three members of genes encoding phosphatidic acid phosphatase showed differential expression patterns: two of them (F0438 and F0553) were up-regulated during infection, but the other (MGG_09330) was down-regulated. F0438 was induced in nitrogen starvation, MGG_09330 in carbon starvation, and all three were down-regulated during conidiation and conidia germination.

### Use of ESTs in validation and improvement of genome annotation

**Figure 5:**
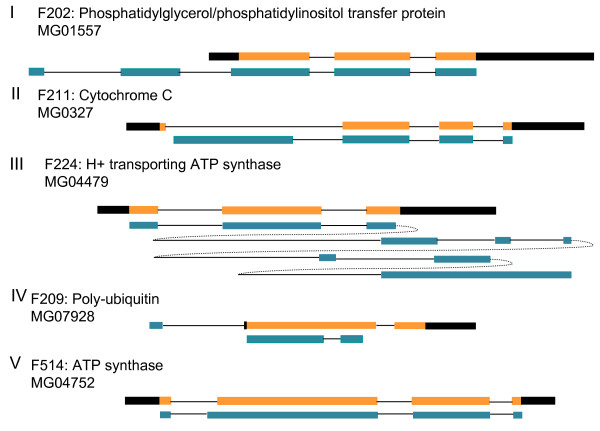
**Five types of errorneous gene model were illustrated**. Orange bar represents exon region translated from cDNA sequences, blue bars represent exon region, black bars 5' and 3' untranslated region, and line intron region, respectively. Extremely long intron was described as dotted line in type III.

ESTs can complement automated genome annotation efforts [33]. 125 uniESTs showed no sequence similarity to *ab initio *annotated gene sequences, but matched the genome assembly. These loci can now be re-annotated as genes. BLASTX results against the NCBI non-redundant protein database were carefully examined for ESTs that matched from a start codon to search for ESTs having full-length cDNA sequences. Among the 712 fungal genes, 83 uniESTs were found to have full-length ORFs through analysis of BLASTX results. These include genes uniquely identified from the infection EST, including genes encoding cytochrome P450 (F0125), co-chaperone (F0132), and alcohol dehydrogenase (F0289) (Additional file 3, Table S3). Representative clones were retrieved, and entire inserts were sequenced using primer walking. It is possible that polymorphisms may be found by comparing the cDNA sequences to the genome, because our cDNA sequences were derived from *M. oryzae *strain KJ201, not strain 70-15, which was used for genome sequencing; however, on average, the two strains are more than 99% identical in exon regions. Of 66 genes compared, eight had single nucleotide polymorphisms, and 10 bases out of 33,198 nucleotides (0.03%) compared were different between two strains in the exon region (data not shown). Next, full-length cDNA sequences were compared with genome sequences comprising each locus and ORF sequences automatically annotated from the genome. The average ORF length translated from cDNA sequences was 559 bases (range, 192-1,506). There were discrepancies between *ab initio *genome annotation and full-length cDNA in 24 of 83 clones (about 28%), suggesting incorrect annotation or the possibility of alternative splicing. There were five types of mis-annotation (Fig 5). One group had long N-terminal extensions in the electronic annotation, compared with coding sequences (CDs) inferred from cDNA sequences. In the second group, electronic annotations revealed relatively short CDs in which the start codon resided in an intron or in the middle of coding regions of cDNA sequences. In four cases, the electronically annotated locus had long C-terminal extensions due to erroneous splicing. Another group of loci started at the same site of cDNA, but had another intron in the cDNA, resulting in short ORFs. These sequences did not seem to be the splicing variant between fungal strains, because ESTs from strain 70-15 in which the genome sequence was revealed matched exactly with our cDNA sequences. In these four cases, electronic annotations started and stopped at the exact sites, but depicted other internal exon-intron boundaries. These analyses showed that, in most cases, erroneous splicing was attributed to mis-annotation. Therefore, the splicing site context of the *M.oryzae *genome was further analyzed using full-length cDNA sequences.

### Splicing site context

**Figure 6:**
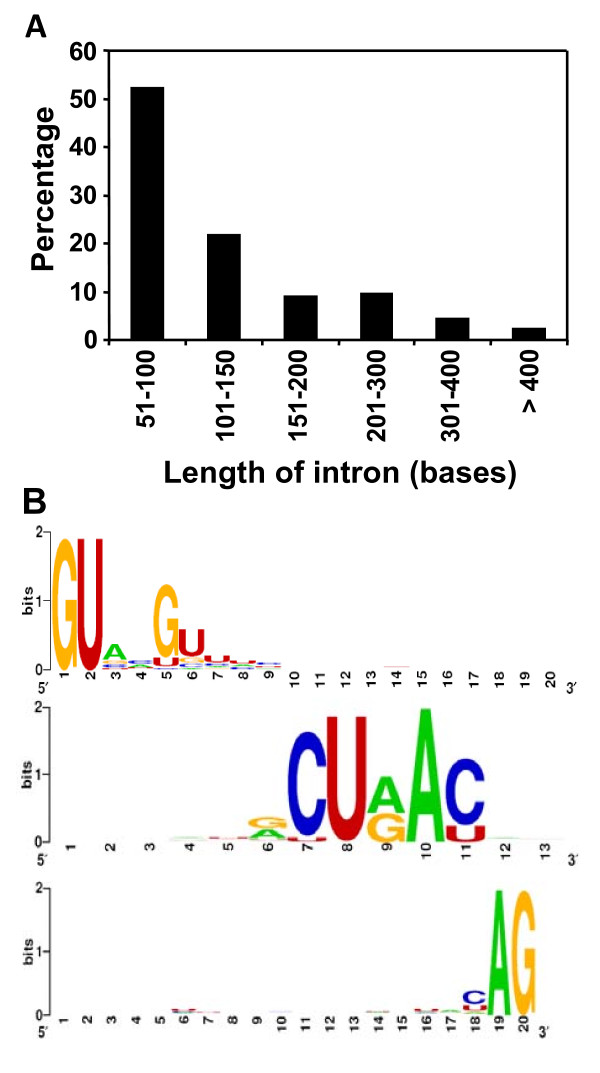
**Intron length distribution and splicing site context**. (A) Intron length distribution of 68 full-length cDNA sequences. (B) Splicing site consensus sequences for 5' exon-intron junctions (upper), branch point (middle), and 3' intron-exon junctions (lower) were calculated using WebLogo server at http://weblogo.berkely.edu. The general consensus sequences of each region were displayed in order of predominance from top to bottom at each position with large letter being higher frequency.

Fungal gene expression, as in higher eukaryotes, accompanies accurate splicing via the coordinated efforts of a spliceosome. *Cis *elements, consisting of the 5'- and 3'-splice sites, have been found universally to be predominantly GU and AG, respectively, the branch point A and surrounding motif, and polypyrimidine tracts [34]. These fl-cDNA sequences were also used to identify accurate exon-intron boundaries and conserved intron *cis *elements in *M.oryzae*. 165 intron sequences from 68 genes were extracted by aligning ESTs to genomic sequences, and these intron sequences with adjacent exon sequences were examined for correct splicing. The mean intron length was 137 nucleotides (range, 53-883, and a dominant peak distribution between 50-100 nucleotides; Fig. 6A). All but one intron examined had the canonical 5'GU...AG3' donor acceptor splicing site pair. The consensus sequences for the region of the 5'-ss, branch site, and 3'-ss are shown as a component of the structure logos in Fig. 6B. The 5'ss consensus sequence was found to be NG|GURMGY, which is more degenerate than NG|GURAGU, a filamentous fungal consensus sequence suggested by Kupfer et al. [34]. The 3'ss consensus sequence was YAG, which is characteristic of metazoans. More than 98% of the introns appeared to have a potential branch site, a key intron element required for lariat formation during the splicing process, according to icat.pl script. The consensus sequences could be derived to be RCURAY, where the underlined A is the branch point. This is consistent with the results of Kupfer et al. [34]. The branch point A could be localized to a position between 5 and 160 nt from the 3'-end of the intron, in which most of them (89.7%) were localized between 11 and 30 nt. Polypyrimidine tracts, another conserved element in mammalian introns, which function as a binding site for spliceosomal protein U2AF^65^, were also found in most introns (94%) screened in this study. About 43% of them were only located from the 5'ss to the branch point, and 45.4% were located on both sides of the branch point. These results are also in agreement with Kupfer et al. [34].

## Discussion

Fungal genes expressed during infectious growth within their host and plant genes expressed after attack by their pathogen may have important role(s) in virulence and defense responses. In this study, we identified 2,302 uniESTs expressed during rice-*M. oryzae *interactions using two complementary approaches. The first approach was EST analysis (IL) of blast fungus-infected rice leaves with rapidly expanding typical blast lesions. We harvested leaves with typical lesions and collected these daily from 410 days after inoculation. 1,539 uniESTs were identified from the EST analysis. The second strategy was using SSH (SL) to enrich fungal genes and rice genes, whose expression was induced during interactions, resulting in the generation of 963 uniESTs.

### Fungal genes expressed during infectious growth were identified

cDNA libraries were constructed with RNA from rice leaves infected with rice blast fungus. Thus, pathogen genes, as well as host plant genes expressed during their interaction, were contained in these libraries. Assigning the origin of ESTs can be complex. The G+C content was successfully used to identify oomycete genes from soybean genes infected with *Phytophthora sojae *[35]; however, this approach is not applicable for pathosystems involving ascomycete fungi. Sequence similarity to previously identified genes is widely used as a major criterion to distinguish their origin [7,11,17,19,20]. However, ESTs that did not show significant sequence similarity to database entries could not be annotated. This ranged from 30% to more than 40% of ESTs, depending on the organism. We could not identify the origin of 42.7% of ESTs in our previous study [7]. Li et al. [19] adapted codon usage pattern as a subsidiary tool to differentiate *Sclerotinia sclerotiorum *genes from *Brassica napus *genes. However, rice and *M. oryzae *genes do not show significant differences in their codon usage pattern when we analyzed full-length cDNA sequences (data not shown). In this study, we took advantage of genome sequences and many EST sequences of both organisms to distinguish the origin of the ESTs. With the BLASTX results against NCBI nr, BLASTN data against genomic and EST sequences of both organisms, 99% of uniESTs could be annotated for their origin (Additional file 1 Table S1). Among these, 712 uniESTs were identified as fungal genes, comprising 31% of the 2,302 uniESTs. 402 uniESTs (533 ESTs) were from the infected library, comprising 24% of the uniESTs (27% of total ESTs), and 310 uniESTs were added from the subtraction library.

A relatively high proportion of ESTs were of fungal origin. This result is consistent with our previous study [7], where about 24.6% of uniESTs were believed to be fungal genes. Interaction transcriptome [36] approaches have been used for understanding molecular mechanisms of defense and/or pathogenicity in a variety of pathosystems [7,11,17-20,37]. However, only a limited number (or proportion) of fungal genes were identified. This is primarily because previous authors used plant material at early stages of infection, or partially resistant host cultivars. Jantasuriyarat et al. [11] analyzed 68,920 ESTs from eight cDNA libraries including six constructed from rice leaves infected with rice blast fungus, and found only four fungal genes. They collected rice leaves at 6 and 24 h after blast fungus inoculation, which are the very early stages of infection when fungal conidia have just germinated or just started to penetrate into rice epidermal cells. Talbot et al. [29] estimated fungal biomass to be 10% at 72 h after inoculation, at which time lesions start to appear. Recovering a high portion of fungal genes in this study might be attributed to the leaf material from which total RNA was isolated. We collected diseased leaves at late stages of infection daily up to 10 days after inoculation, when the whole leaf blade was covered with expanding lesions and had started to wither. Thus, fungal genes found comprised 27% of uniESTs in the infection library; this result was not unexpected. These fungal genes will provide a good resource to understand fungal pathogenicity, especially at later stages of infection.

### Subtraction efficiency is high

43.5% of fungal genes (310 uniESTs of 712 fungal genes) were obtained from a subtraction library. 50% (1,128 of 2,259) of the ESTs from the subtraction library were identified as fungal genes. We used uninfected rice RNA as a driver to enrich fungal genes as well as rice genes that were up-regulated on fungal infection. Gilleroux and Osbourn [20] used two driver cDNA populations (i.e., one derived from mock-inoculated roots and the other from a *G. graminis *culture grown in complete medium) to subtract cDNAs from *G.graminis*-infected wheat roots. However, they did not identify any fungal gene using this strategy, possibly because fungal genes comprise a low proportion of infected root tissue and/or the fungal genes expressed during plant infection are also expressed during growth *in vitro*, to be subtracted out by the fungal driver. Using uninfected plant RNA as a driver turned out to be a useful strategy to identify candidate fungal genes to be functionally characterized. In addition to cataloging fungal genes expressed during infection, we could identify suites of rice genes encoding ABC transporter families specifically obtained from the subtraction library. Forty-two ESTs (18 uniESTs) showed sequence similarity to 11 different ABC transporter families, of which 38 ESTs were from the subtraction library. Seventeen ESTs encoded *PDR9*, expression of which is induced by environmental stresses, such as heavy metals, hypoxic stress, and redox perturbations [38]. Direct evidence for a role of ABC transporters in plant defense was obtained from the study of *NpPDR1 *(formerly named *NpABC1*), a plasma membrane PDR type ABC transporter of *Nicotiana plumbaginifolia*, which was induced by the elicitor analog sclareolide, sclareol, a virulent strain of *Pseudomonas syringe *pv. *tabaci *and by non-pathogenic pseudomonads, *P. fluorescens *and *P.marginalis *pv *marginalis *[39], and is believed to be involved in the secretion of the antimicrobial diterpene, sclareol [40]. Transgenic plants in which *NpPDR1 *expression was prevented by RNA interference showed reduced resistance to the fungal pathogen *Botrytis cinerea *[39]. Thus, ABC transporters identified in this study may play a role in rice defense against the invading fungus. These results lead us to suggest that various materials toxic to plant cells might be excreted from invading fungal cells into the plant cytosol, and that plant cells struggle to pump out and/or detoxify these deleterious materials. This was further supported by the fact that a high proportion (10.4%, 74 genes) of *in planta *expressed fungal genes encoded proteins with a signal peptide.

There was little overlap in the fungal genes identified from the two libraries in terms of uniEST. This may be attributed to the low depth of coverage in this study. Another explanation may be drawn if we consider the number of total ESTs, as most overlapped sequences are highly redundant so that 587 ESTs comprised 75 uniESTs.

### Novel genes identified

Although EST collections form the foundation for various genome-scale experiments within as yet unsequenced genomes [33], they provide valuable information for gene discovery, genome annotation, and expression profiles, even in organisms where the genome sequence is available. They have also been successfully applied to identify genes expressed during the interaction of the plant pathogen with its host [7,35]. Although 12,465 uniESTs of *M. oryzae *from nine different libraries [12] were already available, we found 100 previously unidentified unigenes through analysis with strict criteria. This exactly identified 14% of the 712 fungal genes. Sixty seven unigenes were sequenced from the infection library, while 37 genes were added from the subtraction library. The nine libraries that Ebbole et al. [12] used were constructed with RNA covering the fungal life cycle, including conidia, appressoria forming germlings, and mixed culture undergoing mating, which are stress conditions thought to mimic the environment the fungus might encounter in nature, such as rice cell walls, complete medium, minimal medium, nitrogen starvation, and mixed stress conditions, and mycelia from mutants that could not elaborate the infection structure, the appressorium. Nutrient starvation, especially nitrogen-deprived conditions, is regarded as an environmental cue for the fungus to infect the plant [41,42]. *M. oryzae *secrets proteins that cause senescence of rice leaves, reminiscent of the symptoms caused by the fungus itself when it is under nitrogen starvation [42]. *NPR1 *and *NPR2*, nitrogen-regulatory genes non-allelic to *NUT1*, were reported to positively regulate both nitrogen metabolism and pathogenicity [43]. However, many genes were newly identified only from *in planta *ESTs, indicating that the repertories of genes expressed during infectious growth and under nutrient stress might differ, and that nutrient stress might not fully represent the conditions that the fungus would meet within the plant cell, as suggested in our previous work [7].

### Resources for improvement of genome annotation

ESTs can be used in gene discovery and genome annotation. Of 712 ESTs from this study, 125 (17.5% of fungal ESTs) did not match any electronically annotated gene. These sequences will help in further correction of genome annotations. Validation of genome annotation could further be confirmed by comparing full-length cDNA sequences with genome sequences. Full-length or near full-length cDNA encompassing the complete ORF can serve as a valuable resource for accurate genome annotation and functional analysis. Large-scale full-length cDNA sequencing projects with the aim of identifying the complete transcriptome have been conducted and used for improving genome annotation in many model organisms, including *Arabidopsis thaliana *[44-47] and rice [25], as well as humans [48] and mice [49,50]. For example, 32% of the first version of the *Arabidopsis *gene model was found to be inaccurately annotated, of 10,507 genes having fl-cDNA sequences, and was improved by incorporation of fl-cDNA sequences [47]. The rice genome community has recognized the value of fl-cDNA sequences and deep EST resources, and acquired more than 28,000 fl-cDNA clones prior to completion of the genome sequence [25]. Few efforts have been made to collect and use full-length cDNA sequences in filamentous fungi, in which targeted gene deletion mutants could readily be obtained by homologous recombination due to the haploid nuclear status. However, full-length cDNA sequences from filamentous fungi still have the same significance for accurate genome annotation, functional analysis, and for proteomics as in other eukaryotes, because fungal genomes are also interspersed with non-coding DNA sequences. In this respect, full-length cDNA clones acquired in this study could be a valuable resource for the community. Of 83 full-length cDNAs, 24 (28.6%) showed different gene structures from the *ab initio *annotated gene model. Electronic annotation started at different positions in 11 cases and erroneously stopped in eight cases, resulting in longer or shorter ORFs. In the remaining cases, electronic annotation started and stopped at the exact sites, but depicted erroneous internal exonintron boundaries. In most cases, erroneous splicing was attributed to the wrong gene model. The intron *cis *elements (i.e., 5'-GU...AG-3' donor-acceptor splicing site pairs, 5'- and 3'-ss consensus sequence, internal branch site, and polypyrimidine tracts) were also conserved in the *M. oryzae *genome, and were consistent with results for other filamentous fungi, with more degenerate 5'-splice sites, as suggested by Kupfer et al. [34]. These results can be used to further improve genome annotation.

In summary, genome-wide identification of genes expressed during the interaction between rice and the invading fungal pathogen *M. oryzae *was carried out and resulted in cataloging of 2,302 uniESTs. More than 700 fungal genes were identified, of which 100 genes were newly identified in this study, and more than 80 genes with full-length ORF sequences were used to validate the genome annotation. Further characterization of the genes reported here will help to unravel the mechanisms of pathogenicity, especially at later stages of infection, as well as the defense responses of the host plant.

## Conclusions

Infection of plants by pathogens and the subsequent disease development are accompanied by substantial changes in the physiology of both partners. Analysis of genes expressed during these interactions represents a powerful strategy to obtain insights into the molecular events underlying these changes. Here, we used two complementary strategies, EST and SSH, to catalog genes during a compatible rice and blast fungus interaction at late stages of infection. Fungal genes constituting as many as 30% of uniESTs, as well as plant defense genes, were identified. We identified 100 previously unreported fungal genes despite the wealth of genome resources on this fungus. Additionally, whole sequences from clones having full-length cDNAs were determined by primer walking, which was used to verify the *ab initio *annotated gene model of *M. oryzae *genome sequences and splicing context analysis. Taken together, the data obtained in this study will serve as a valuable resource to expand our understanding of genome organization and molecular aspects during rice *M. oryzae *interactions.

## Methods

### Construction of cDNA libraries

Rice plants (*Oryza sativa *L. cv. Hwacheong) at the 3^rd ^to 4^th ^leaf stages were spray-inoculated with 2 × 10^5 ^spores/ml. After keeping in a dew chamber for 24 h at 25°C, the inoculated plants were transferred to the greenhouse to let the lesions develop. Rice leaves on which typical susceptible type lesions spread through the entire leaves were cut and immediately frozen in liquid nitrogen daily up to 10 days after inoculation (*in planta *sample). Total RNA was isolated from the frozen plant tissues through phenol/chloroform extraction, followed by lithium chloride precipitation [51]. Poly A(+) RNA was purified using the PolyATrack mRNA isolation system (Promega) and used for construction of two libraries. cDNA was synthesized using the lamda ZAP cDNA synthesis kit and cDNAs larger than 0.5 kb after gel filtration were used to construct the cDNA library (infection library: IL) using the ZAP Express cDNA Gigapack III Gold Cloning kit (Stratagene, La Jolla, CA, USA). Mass *in vivo *excision was conducted to rescue phagemid clones. Individual colonies were grown in LB medium amended with 100 μg/ml ampicillin in 96 well plates and used for plasmid preparation. A subtracted cDNA library was also constructed by suppression subtractive hybridization using PCR-select™ cDNA subtraction kit (Clontech, Palo Alto, CA, USA) following the manufacturer's instructions. cDNA obtained from *M. oryzae *infected rice leaves was used as a 'tester' and cDNA from uninfected healthy leaves was used as a 'driver'. Subtracted PCR products were cloned into pCR4 blunt-TOPO vector (Invitrogen) and transformed into *E. coli *strain TOP10 (Invitrogen) with blue-white selection. White colonies were grown in LB plates amended with ampicillin and used for plasmid preparation.

### EST sequencing and analysis

Plasmid DNAs were purified from 1 ml each of the overnight grown bacterial cultures and subjected to automated sequencing using ABI PRISM BigDye Terminator on ABI Prism 3700 sequencer (Applied Biosystems, Foster City, CA, USA) or using ET terminator dye (Amersham, Uppsala, Sweden) on the multicapillary sequencer of RISA384 system (Shimadzu, Tokyo, Japan). Sequence analysis was conducted with an automated pipeline embedded in the web-based bioinformatic systems, Comparative Fungal Genomics Platform (http://cfgp.snu.ac.kr) [52] and Fungal EST Database (FED; http://fedb.snu.ac.kr, Park et al., in preparation). Sequence data in the chromatogram file were processed using Phred [53] to call bases with cutoff value >20 and Crossmatch to mask vector sequences. The output sequences were further trimmed using a computer script to get rid of the poly A or poly T tracks. The sequences longer than 100 bp were collected and clustered using CAP3 [54]. The resulting uniESTs were searched against GenBank nr database using a BLASTX algorithm, and against rice [3,4] and *M. oryzae *[2] genome sequences with the BLASTN algorithm. Functional categorization was conducted with GO term assignment. *Ab initio *annotated ORFs from *M. oryzae *genome and the translated rice full-length cDNA sequences, which have corresponding infectionESTs were used to search the InterPro database (version12), and the InterPro terms were mapped to GO terms as described in Ebbole et al. [12]. For full-length cDNA sequences, representing cDNA clones were retrieved and their sequences read by primer walking. The intron and exon databases were created by using FELINES [55] with the parameters used by Kupfer et al. [34]. Splicing site consensus sequences for 5' exon-intron junctions, branch point, and 3' intron-exon junctions were calculated using WebLogo server at (http://weblogo.threeplusone.com/)[56]. EST sequences were deposited into dbEST of NCBI under the serial accession number from GT930449 to GT934681, GT968229, and GT968230. The full length cDNA sequences were deposited into NCBI under the serial accession numbers from GU395207 to GU395289 and listed in Table S3.

### Real-time RT-PCR

Real-time RT-PCR was conducted following the protocols described previously [8] with slight modification. Total RNA for cDNA library was used to assess the expression level during infectious growth. Fungal tissue for expression dynamics during development and nutrient starvation was prepared according to the procedures as described previously [8]. Conidia bearing mycelia grown on oatmeal agar for 10 days at 25°C were harvested by scraping the surface with razor blade. A total of 2 × 10^7 ^conidia were incubated in liquid complete media for 8 hours at 25°C by shaking to get germinated conidia. Mycelia for nutrient starvation conditions were prepared as described in Talbot et al. [29]. Samples were frozen in liquid nitrogen immediately after harvest, ground with pre-chilled mortar and pestle, and stored at -80°C before use. Total RNA was isolated from frozen mycelial powder using an Easy-Spin RNA extraction kit (iNtRON Biotechnology, Seoul, Korea). Five micrograms of total RNA was reverse transcribed into first-strand cDNA with oligo (dT) primer using SuperScript™ First-Strand Synthesis System (Invitrogen, Carlsbad, CA, USA) according to the manufacturer's instruction, and diluted to the concentration of input RNA to be 12.5 ng/μl with nuclease-free water. Reactions were done in a 10 μl volume containing 100 nM of each primer, 2 μl cDNA (25 ng of input RNA) and 5 μl of 2× iQ™ SYBR^® ^Green Supermix (Bio-rad, Hercules, CA, USA). Real-time PCR was run on an iCycler iQ Real-Time PCR Detection System (Bio-Rad, Hercules, CA, USA). After 3 min denaturation at 95°C, samples were run for 40 cycles of 15 s at 95°C, 30 s at 60°C, and 30 s at 72°C. After each run, amplification specificity was checked with a dissociation curve acquired by heating the samples from 60 to 95°C. Relative abundance of transcripts in complete media was calculated with 2^-ΔCt^, where ΔCt = (C_t, gene of interest_- C_t, cyclophilin_)_CM_. Fold changes during infectious growth and growth under nutrient starvation compared to growing in liquid complete medium were calculated with 2^-ΔΔCt^, where ΔΔCt = (C_t, gene of interest_- C_t, cyclophilin_)_test condition _- (C_t, gene of interest _- C_t, cyclophilin_)_control _[57]. Real-time PCR was conducted with 3 replicates. The primer pairs for transcripts amplification were denoted in Additional file 4 (Table S4).

## Authors' contributions

SK and YH designed the experiments and wrote the manuscript. SK also carried out the experiments and analyzed the data. SP participated in full-length cDNA sequencing, JP did bioinformatics and developed the database. TKM participated in discussion and manuscript preparation. All authors read and approved the final manuscript.

## Supplementary Material

Additional file 1 **List of abundant ESTs sequenced more than 20 times**. List of genes frequently represented in each library. Click here for file

Additional file 2 **List of genes specific to infection EST**. List of novel genes uniquely or preferentially expressed during rice-*M. oryzae *interactions. These were identified by the comparing the publically available genomic resources as illustrated in Fig. 3. Click here for file

Additional file 3 **List of genes with full length cDNA**. cDNAs with full-length ORF and their accession number were presented. Click here for file

Additional file 4 **Primer information for genes used in real-time RT-PCR**. List of primers used in real-time RT-PCR. The expression data was presented in Fig. 4. Click here for file
