# Supplementary material for: Identification and analysis of in planta expressed genes of Magnaporthe oryzae
Source: BMC Genomics. 2010 Feb 10;11:104. doi: 10.1186/1471-2164-11-104 (PMC2832786; doi:10.1186/1471-2164-11-104)
Supplement: Additional file 1 — List of abundant ESTs sequenced more than 20 times. List of genes frequently represented in each library. [file 1471-2164-11-104-S1.DOC]

Table S1. List of abundant ESTs sequenced more than 20 times

Infection library Subtraction library

Contig Putative identification (GI) No. Origin^a^ Contig Putative identification (GI) No. Origin^a^

Contig526 Rubisco activase (8918359) 22 P Contig203 EF1-alpha (729395) 76 F

Contig106 Fructose-bisp aldolase (3913018) 14 P Contig226 No hit 63 F

Contig496 Metallothionein (7489497) 13 P Contig200 Senescence-associated protein (13359451) 29 P

Contig519 Pyruvate dehydrog. kinase1 (12829952) 11 P Contig346 Isocitrate lyase (113026) 24 P

Contig549 Rubisco small subunit C (132105) 9 P Contig474 No hit 24 F

Contig310 Hypothetical protein (19115215) 8 F Contig135 Rpl9bp (6324262) 21 F

Contig370 Glucose-repressible gene (121613) 8 F Contig173 Probenazole-induced protein (7442204) 20 P

Contig597 PR1 (7442184) 8 P Contig153 Metallothionein I (2497883) 19 P

Contig561 translation factor SUI1 (462195) 7 P Contig550 Cytochrome C (118009) 19 F

Contig566 ATP-dependent Clp protease (18423233) 7 P Contig415 No hit 18 P

Contig602 Hypothetical protein (19113391) 7 F Contig562 CPEP phosphonomutase (6831518) 17 P

Contig76 PSII 10K protein (7443194) 7 P Contig134 No hit 16 F

^a^ Putative origin of the ESTs were indicated. P, plant gene; F, fungal gene.
