# Supplementary material for: Identification and analysis of in planta expressed genes of Magnaporthe oryzae
Source: BMC Genomics. 2010 Feb 10;11:104. doi: 10.1186/1471-2164-11-104 (PMC2832786; doi:10.1186/1471-2164-11-104)
Supplement: Additional file 2 — List of genes specific to infection EST. List of novel genes uniquely or preferentially expressed during rice-M. oryzae interactions. These were identified by the comparing the publically available genomic resources as illustrated in Fig. 3. [file 1471-2164-11-104-S2.DOC]

Table S2. List of genes specific to infection EST

ID MG Locus GI number Blast Best Hits Organism E-value

F0031 MGG_02886 No hit

F0034 MGG_06397 6324227 Pol1p *S. cerevisiae* 7.00E-22

F0066 MGG_04205 1022722 avenacinase *G. graminis* 3.00E-09

F0069 No hit

F0071 MGG_09852 No hit

F0072 MGG_05383 No hit

F0095 MGG_10791 No hit

F0125 MGG_06047 19114700 cytochrome p450 *S. pombe* 4.00E-48

F0127 MGG_11382 6320296 RNase L Inhibitor (Rli1p) *S. cerevisiae* 1.00E-09

F0151 9294588 Ser-Thr protein kinase-like *A. thaliana* 4.00E-06

F0170 MGG_02582 No hit

F0179 MGG_02739 7293987 CG3877 gene product *D. melanogaster* 3.00E-07

F0186 MGG_06227 No hit

F0189 MGG_03572 19115684 signal recognition particle *S. pombe* 1.00E-05

F0199 MGG_06862 No hit

F0240 MGG_12093 6324296 Siw14p *S. cerevisiae* 2.00E-29

F0244 MGG_02739 19114042 ubiquinone biosynthesis protein *S. pombe* 5.00E-36

F0246 MGG_06862 No hit

F0247 MGG_04946 13476699 Hypothetical protein *M. loti* 2.00E-05

F0248 No hit

F0254 No hit

F0255 MGG_03956 18376087 TFIID subunit TAF19 *N. crassa* 4.00E-12

F0256 MGG_05683 No hit

F0263 MGG_08801 17136406 pn-P1 *D. melanogaster* 1.00E-09

F0273 MGG_03321 6320735 Protein for cell viability; Ydr527wp *S. cerevisiae* 2.00E-12

F0279 MGG_04718 No hit

F0280 No hit

F0283 No hit

F0285 MGG_02712 19113575 adenosine deaminase *S. pombe* 1.00E-12

F0287 MGG_11913 18375999 mannosylphospho. protein MNN4 *N. crassa* 2.00E-25

F0295 MGG_11408 5107145 alpha-mannosidase *C. dubliniensis* 1.00E-06

F0303 No hit

F0305 MGG_09738 534844 beta-glucosidase *C. wickerhamii* 9.00E-44

F0307 MGG_01348 No hit

F0311 MGG_01608 6322484 Hypothetical ORF; Yjr024cp *S. cerevisiae* 1.00E-21

F0312 MGG_12119 11595728 hypothetical protein *N. crassa* 9.00E-36

F0319 MGG_11818 16415978 2, 5-DKGR *N. crassa* 2.00E-44

F0332 No hit

F0341 MGG_13455 16944622 DFG5 protein *N. crassa* 3.00E-11

F0343 No hit

F0359 MGG_13472 14269413 histidine-tRNA ligase *O. novo-ulmi* 5.00E-41

F0366 MGG_03546 No hit

F0371 MGG_08845 No hit

F0375 MGG_13672 No hit

F0382 No hit

F0393 MGG_06062 1197523 nitrate reductase *B. fuckeliana* 6.00E-05

F0396 MGG_05682 2494623 G protein pathway suppressor 1 *H. sapiens* 8.00E-15

F0399 MGG_13520 No hit

F0415 MGG_10320 12965187 methylcrotonoyl-CoA carboxylase *M.musculus* 2.00E-10

F0417 No hit

F0427 No hit

F0428 MGG_13963 No hit

F0438 MGG_05988 16943771 phosphatidic acid phosphatase *P. ostreatus* 3.00E-28

F0440 MGG_02120 No hit

F0444 MGG_00967 12963344 Tid56-like protein *M. musculus* 3.00E-14

F0445 MGG_04179 19113831 ATP-dependent RNA helicase *S. pombe* 2.00E-45

F0446 No hit

F0448 No hit

F0461 MGG_04682 No hit

F0468 No hit

F0478 MGG_04842 2492647 Homoaconitase *E. nidulans* 2.00E-58

F0480 MGG_06322 No hit

F0494 MGG_08683 No hit

F0501 No hit

F0505 MGG_02773 2708784 MADS-box homolog Umc1 *U. maydis* 8.00E-27

F0507 MGG_00473 No hit

F0509 MGG_09205 No hit

F0518 MGG_09418 No hit

F0521 MGG_07089 6321016 tRNA nucleotidyltransferase *S. cerevisiae* 3.00E-25

F0522 No hit

F0536 MGG_04321 No hit

F0538 MGG_05218 No hit

F0541 MGG_09223 15894807 Beta-xylosidase *C.acetobutylicum* 8.00E-10

F0546 MGG_01632 No hit

F0553 MGG_09994 16943771 phosphatidic acid phosphatase *P. ostreatus* 1.00E-18

F0554 MGG_03526 13940380 hypothetical protein *Z. rouxii* 1.00E-13

F0559 MGG_11237 No hit

F0561 464381 3-phytase A *A. awamori* 6.00E-24

F0564 MGG_06366 19115509 zinc finger-like protein tim13 *S. pombe* 2.00E-14

F0566 MGG_09852 6320745 sugar transporter-like protein; Stl1p *S. cerevisiae* 6.00E-30

F0573 MGG_05988 No hit

F0575 MGG_02418 19115602 sec18 homolog *S. pombe* 2.00E-24

F0576 MGG_12773 15598135 aminopeptidase *P. aeruginosa* 3.00E-12

F0580 MGG_09418 18376320 hypothetical protein *N. crassa* 4.00E-13

F0584 MGG_07443 3023397 oxidoreductase bli-4 *N. crassa* 3.00E-07

F0597 MGG_06489 17935455 alcohol dehydrogenase *A. tumefaciens* 3.00E-12

F0601 No hit

F0607 MGG_04863 13278966 Unknown *H. sapiens* 3.00E-18

F0608 MGG_00874 19114968 dam1 *S. pombe* 1.00E-12

F0622 MGG_13969 No hit

F0623 MGG_10857 7493925 apsB protein *E. nidulans* 2.00E-38

F0633 MGG_08079 18376306 hypothetical protein *N. crassa* 5.00E-31

F0636 MGG_10724 5545331 fatty acid omega-hydroxylase *F. oxysporum* 3.00E-

F0646 No hit

F0648 No hit

F0663 No hit

F0677 No hit

F0679 No hit

F0691 No hit

F0704 MGG_05082 No hit
