# Supplementary material for: Identification and analysis of in planta expressed genes of Magnaporthe oryzae
Source: BMC Genomics. 2010 Feb 10;11:104. doi: 10.1186/1471-2164-11-104 (PMC2832786; doi:10.1186/1471-2164-11-104)
Supplement: Additional file 3 — List of genes with full length cDNA. cDNAs with full-length ORF and their accession number were presented. [file 1471-2164-11-104-S3.DOC]

Table S3. List of genes with full length cDNA

F_No Locus Accession Putative identification Organism E-value

F0001 MGG_06185 GU395207 acidic ribosomal protein P1 *Aspergillus fumigatus* e-150

F0002 MGG_09194 GU395208 60S ribosomal protein L17 *Neurospora crassa* 0

F0003 MGG_04696 GU395209 60s ribosomal protein l10a. *S. pombe* 0

F0004 MGG_04114 GU395210 40S ribosomal protein S17 (CRP3) *N. crassa* 0

F0017 MGG_02711 GU395211 60S ribosomal protein L24 (L30) *A. fumigatus* 2e-45

F0019 MGG_02872 GU395212 ribosomal protein S27 *S. frugiperda* e-140

F0033 MGG_03680 GU395213 Rps11bp *S. cerevisiae* 0

F0039 MGG_03727 GU395214 60s ribosomal protein *C. gloeosporioides* 0

F0046 MGG_09736 GU395215 hypothetical serine-rich protein *S. pombe* 4e-53

F0070 MGG_04104 GU395216 60s ribosomal protein l22 *S. pombe* 0

F0098 MGG_06000 GU395217 hypothetical protein *S. pombe* 0

F0110 MGG_06019 GU395218 Initiation factor 5A (EIF-5A) *N. crassa* 0

F0113 MGG_03135 GU395219 ribosomal protein L30 *O. novo-ulmi* 0

F0115 MGG_01679 GU395220 Conserved hypothetical protein *S. meliloti* 0

F0121 MGG_00715 GU395221 Glucose-repressible gene protein *N. crassa* e-120

F0132 MGG_06669 GU395222 Hsp90 associated co-chaperone *N. crassa* 2e-44

F0140 MGG_04489 GU395223 Nonhistone chromo. protein 6B *S. cerevisiae* e-174

F0148 MGG_13474 GU395224 transl. initiation factor 3 (Sui1p ) *S. cerevisiae* 0

F0152 MGG_02710 GU395225 Peroxisomal membrane PMP20 *A. fumigatus* 0

F0189 MGG_03572 GU395226 signal recognition particle 14 kda *S. pombe* 0

F0193 MGG_06249 GU395227 transl. controlled tumor protein *S. pombe* 0

F0195 MGG_05449 GU395228 probable ribosomal protein l13a *N. crassa* 0

F0196 MGG_02392 GU395229 ribosomal protein s30 *S. pombe* e-106

F0201 MGG_10680 GU395230 Homology to rat S24; Rps24ap *S. cerevisiae* 0

F0202 MGG_01557 GU395231 phosphatidylgly/phosphatidylino transfer protein *A. oryzae* 0

F0209 MGG_07928 GU395232 poly-ubiquitin *M. grisea* 0

F0210 MGG_06658 GU395233 40S ribosomal protein S5 *C. arietinum* 0

F0211 MGG_06685 GU395234 Cytochrome C *N. crassa* e-112

F0214 MGG_02659 GU395235 Homology to mammalian L14 *S. cerevisiae* 0

F0215 MGG_10497 GU395236 BLI-3 protein *N. crassa* 0

F0216 MGG_05238 GU395237 ribosomal protein S14.e *N. crassa* 0

F0217 MGG_07929 GU395238 40S ribosomal protein S26E *N. crassa* 0

F0220 MGG_08203 GU395286 yeast mbf1 homolog *S. pombe*

F0222 MGG_03251 GU395239 ribosomal protein Rps8bp *N. crassa* 0

F0224 MGG_13815 GU395240 H+-transporting ATP synthase *E. nidulans* 0

F0225 MGG_06919 GU395241 40S ribosomal protein S3AE *C. albicans* 0

F0228 MGG_12095 GU395242 dihydroflavonol 4-reductase Gre2p *S. cerevisiae*

F0230 MGG_09955 GU395243 hypothetical protein *S. pombe* 0

F0233 MGG_01173 GU395244 magnaporin *M. grisea* e-175

F0235 MGG_13782 GU395245 60S ribosomal protein L19B *S. pombe* 0

F0238 MGG_10315 GU395246 MPG1 *M. grisea* 0

F0252 MGG_02742 GU395247 putative protein *N. crassa* e-118

F0253 MGG_09952 GU395287 gtp-binding nuclear protein spi1. *S. pombe* 0

F0257 MGG_00788 GU395248 Hypothetical ORF; Ylr099w-ap *S. cerevisiae* 0

F0281 MGG_06933 GU395249 calcineurin subunit B *N. crassa*

F0284 MGG_01585 GU395288 peripheral-type benzodiazepine *S. pombe* e-129

receptor

F0289 MGG_00270 GU395250 alcohol dehydrogenase *S. pombe* 0

F0291 MGG_05062 GU395289 conserved hypothetical protein *S. pombe* 0

F0293 MGG_04660 GU395251 cyclin-dependent protein kinase *S. schenckii* 0

F0294 MGG_07528 GU395252 imidazoleglycerol-phosphate *S. pombe* 0

dehydratase (IGPD)

F0309 MGG_02597 GU395253 hypothetical protein *S. pombe* 0

F0317 MGG_06192 GU395254 Ubiquinol-cytochrome C reductase *N. crassa* 0

F0325 MGG_00221 GU395255 40S ribosomal protein S7 *N. crassa* 0

F0328 MGG_02479 GU395256 Nuclear transport factor 2 (NTF-2) *N. crassa* 0

F0345 MGG_06837 GU395257 40S ribosomal protein S21 (CRP7) *S. pombe* e-149

F0346 MGG_04484 GU395258 60S ribosomal protein L18 *S. pombe* 0

F0354 MGG_01315 GU395259 hypothetical protein *S. pombe*

F0360 MGG_06764 GU395260 guanylate kinase *S. pombe* 0

F0372 MGG_01675 GU395261 hypothetical protein *S. pombe* 0

F0373 MGG_04504 GU395262 hypothetical protein B24P7.10 *N. crassa* 0

F0380 MGG_09977 GU395263 ubiquitin-conj. enzyme; Ubc6p *S. cerevisiae* 0

F0489 MGG_04921 GU395264 ribosomal protein L23 *M. musculus* 0

F0496 MGG_06747 GU395265 glutathione S-transferase *B. fuckeliana*

F0514 MGG_04752 GU395266 ATP synthase; Atp4p *S. cerevisiae* 0

F0520 MGG_00620 GU395267 Similar to CGI-14 protein *H. sapiens*

F0524 MGG_03236 GU395268 40S ribosomal protein S6 *N. crassa* 0

F0526 MGG_01062 GU395269 probable autophagy protein AUT7 *N. crassa* 0

F0533 MGG_08078 GU395270 related to tropomyosin TPM1 *N. crassa* 0

F0551 MGG_03965 GU395271 probable ribosomal protein L26 *N. crassa*

F0554 MGG_03526 GU395272 hypothetical protein *Z. rouxii* 0

F0560 MGG_07753 GU395273 Homology to rat L11; Rpl11ap *S.cerevisiae* 0

F0564 MGG_06366 GU395274 small zinc finger-like tim13 *S. pombe* 0

F0574 MGG_10370 GU395275 40S ribosomal protein S15 (S12) *N. crassa* e-157

F0579 MGG_09006 GU395276 NADH-ubiquinone oxidoreductase *N. crassa* 0

F0580 MGG_09418 GU395277 hypothetical protein *N. crassa* 0

F0582 MGG_06095 GU395278 cysteine dioxygenase *G. mirabilis*

F0590 MGG_08908 GU395279 RIKEN cDNA 2610012O22 gene *M.musculus* 0

F0595 MGG_06895 GU395280 probable ribosomal protein L12 *N. crassa* 0

F0600 MGG_07504 GU395281 NADH-ubiquinone oxidoreductase *S. cerevisiae* 0

F0606 MGG_08597 GU395282 Hypothetical ORF; Ymr099cp *S. cerevisiae*

F0629 MGG_09289 GU395283 hexokinase *A. niger*

F0631 MGG_01070 GU395284 conserved hypothetical protein *N. crassa* 0

F0633 MGG_08079 GU395285 conserved hypothetical protein *N. crassa* 0
