# Supplementary material for: Identification and analysis of in planta expressed genes of Magnaporthe oryzae
Source: BMC Genomics. 2010 Feb 10;11:104. doi: 10.1186/1471-2164-11-104 (PMC2832786; doi:10.1186/1471-2164-11-104)
Supplement: Additional file 4 — Primer information for genes used in real-time RT-PCR. List of primers used in real-time RT-PCR. The expression data was presented in Fig. 4. [file 1471-2164-11-104-S4.DOC]

Table S4. Primer information for genes used in real-time RT-PCR

ID Putative identification Forward primer Reverse primer Amplicon length

F0066 Avenacinase CGCGGTACTGTGAACGTAAA CTGGACTGGGTTGGAGTTGT 172

F0071 No hit ATGGCAGTGACGAAGAAAGC AAACATGCCCGTCTATGGAG 231

F0125 Cytochrome P450 GGTTGACGCAATGACTACGA GGACAATCTTGGGCACTGTT 221

F0189 Signal recognition particle AAGGCGAAGAAGAGGAAAGG CAGTCGTTCTGGGACCTGAT 241

F0244 Ubiquinone biosynthesis protein GTTCCCTCTCCGAAAACACA TTGCAAACTCAAATGCCTTG 219

F0285 dsRNA specific adenosine deaminase TCTGACTCGGCAACAACAAC GAGAGACGAGCAGCGAAGTT 238

F0307 No hit CAACAACATCAACCCACAGC CTCCACCGTCAGGGTTCTAA 214

F0366 No hit TGGCAGCAGTAAGGAGAGGT TTGCGACCACTTGCTGATAG 195

F0438 Phosphatidic acid phosphatase CGAGGGGTGGTAGTTGCTAA CAAGATGGGCAACAACAATG 199

F0445 ATP-dependent RNA helicase TCGACATGGAACTTTGTTGC TGCGCTTCTTCACATATTGC 180

F0505 MADS-box homolog Umc1 CCAAGAAAACCGAGGCATTA CCAGTGAGGACGGATAGCTC 195

F0553 Phosphatidic acid phosphatase ACGCAAAGCTCAAGGTGTTT ATGTGGTTGGTACGCCAGTT 223

F0561 3-phytase A TCAGCACCATCGGATTATGA CGGTACGTGAGGTTTGGAGT 154

F0576 Aminopeptidase CTCTGGGGCTCCAAGTTCTA GGCTTGTTGATGACCTGCAT 272

F0608 Yeast dam1 maintenance TTCAGATCATGCACGAGAGC ATAAAAGTCGCATCCGCATC 208

F0622 No hit GCAAAAGGTAGCCAGTCTCG ACCAAAGGGACCTTTTCTGG 191

F0636 Fatty acid omega-hydroxylase AACCAGCGCCTGTTTGATAG TTGTTCTTGATTGGGTGCAG 167

F0694 GTPase Rho1 GACTCCTCCCAGATCCTGAA TAGTGCCAACTCAACGTGCT 202

MG09330 Phosphatidic acid phosphatase GGGAGGCCTGGATAAGACTC TGTCTACTGTTCCCCGATCC 228
